# The New Abertillery and District Hospital

**Published:** 1920-10-23

**Authors:** 


### The New Abertillery and District Hospital.

Near the top of the mountain above Abertillery is the site of the new hospital for the colliers of the Abertillery district, the foundation-stone of which has now been laid by Sir John Benyon. After many years discussion Councillor T. H. Mytlar and his committee secured unity four years ago, and the colliers began regular subscriptions. A site was bought for £1,100, and Mr. Walter Rosser, of Newport, drafted the plans.

At the ceremony of laying the stone, at which Mr. William Davies, Vice-Chairman of the Hospital Committee, presided, Sir John Benyon said that the new hospital would relieve the Royal General Hospital of much of the work with which it was overburdened. The companies for which he spoke would subscribe £5,000 as a donation, and, in addition, a similar sum, representing 25 per cent. of the sum subscribed by the workpeople hitherto. Further, the companies would be pleased to subscribe 25 per cent. of the amount contributed by the workpeople in the three collieries named on the stone.

Mr. Mytlar remarked that the men had contributed £28,000, and that the hospital was now certain to be built without an overdraft, and that future contributions would go to equip the building. The cost of that is expected to be £50,000, and the hospital will consist of an administration block, two wards of sixteen beds apiece, four wards with single beds, a theatre, and an *x*-ray department.

